# Effectiveness and safety of interventions for fever‐associated discomfort in children: A systematic review

**DOI:** 10.1002/bcp.70203

**Published:** 2025-08-22

**Authors:** Antonio Corsello, Ilaria Alberti, Sara Farhanghi, Alessia Bonetti, Silvia Garattini, Anna Comotti, Paola Marchisio, Elena Chiappini, Gregorio Paolo Milani

**Affiliations:** ^1^ Department of Clinical Sciences and Community Health Università degli Studi di Milano Milan Italy; ^2^ Pediatric Unit Fondazione IRCCS Ca' Granda Ospedale Maggiore Policlinico Milan Italy; ^3^ Department of Health Sciences, Section of Paediatrics University of Florence Florence Italy; ^4^ Occupational Health Unit Fondazione IRCCS Ca' Granda Ospedale Maggiore Policlinico Milan Italy; ^5^ Paediatric Infectious Disease Unit IRCCS Anna Meyer Children's Hospital Florence Italy

**Keywords:** children, discomfort, fever, ibuprofen, paracetamol, systematic review, tepid sponging

## Abstract

**Aims:**

Fever is one of the most frequent reasons for paediatric consultations. While traditionally managed by reducing body temperature, recent guidelines emphasize alleviating discomfort as the primary therapeutic goal. Although different interventions have been described to manage fever‐associated discomfort in children, their effectiveness and safety has never been systematically analysed. The aim of this study was to review the evidence on the effectiveness and safety of pharmacological and nonpharmacological interventions for managing discomfort in febrile children.

**Methods:**

A systematic review was conducted following PRISMA guidelines (PROSPERO: CRD420250655721). PubMed, Embase and Cochrane Library were searched up to 31 January 2025, for studies involving children aged 29 days to 18 years that assessed interventions for fever‐associated discomfort. Randomized controlled trials and observational studies were included. Risk of bias was assessed using Cochrane and STROBE tools. Results were synthesized narratively and grouped according to the type of intervention.

**Results:**

Eight studies (5 randomized controlled trials, 3 observational) involving 1877 children were included. Study designs, including dosage of antipyretics and quality varied across studies. Studies comparing ibuprofen and paracetamol provided conflicting results, while combination therapy (paracetamol + ibuprofen) appeared more effective than using a single drug in ‐one trial. Tepid sponging, despite reducing temperature, was associated with increased discomfort. No serious adverse events were reported.

**Conclusion:**

Pharmacological treatments appear effective and safe, whereas physical methods offer limited benefit. The available evidence is limited by the small number of studies, methodological heterogeneity, and concerns about risk of bias and outcome measurement inconsistency. New high‐quality studies are needed to guide clinical practice for the management of fever‐associated discomfort in children.

## 1 INTRODUCTION

Fever is one of the most common clinical conditions in children, and despite being a natural host‐defence mechanism, it might lead to significant discomfort and distress in children.^1^ Traditionally, the primary goal in managing fever has been to lower the body temperature; however, recent evidence and guidelines suggest that the alleviation of discomfort should be the main target of therapeutic interventions.^2^ Nonetheless, the concept of *discomfort* remains poorly defined, with considerable heterogeneity in its measurement and management across studies.

While pain can be measured with validated scales, it represents only one component of the broader experience of discomfort in febrile children.^3^ Discomfort encompasses a complex set of subjective and observable features, which is not limited to painful experience and that collectively influence the child's wellbeing and caregiver concern.^3^ As such, focusing on discomfort reflects a more holistic, guideline‐recommended approach to fever management. Despite the challenges in its operationalization, studying discomfort as an outcome provides a better alignment with the clinical goal of improving the child's overall comfort and functioning, rather than simply achieving a numeric temperature reduction. Several intervention studies have been conducted to evaluate discomfort management, including pharmacological approaches and physical methods, however their effectiveness has never been systematically analysed.^4^, ^5^ This systematic review aims to summarize the current evidence on interventions for fever‐related discomfort, to compare their effectiveness and to evaluate their safety.

## 2 METHODS

### 2.1 Search strategy

This systematic review was conducted according to the Preferred Reporting Items for Systematic Reviews and Meta‐Analyses (PRISMA) guidelines and has been registered in the International prospective register of systematic review (PROSPERO: CRD420250655721).^6^ A comprehensive literature search was performed in PubMed/MEDLINE, Cochrane Library and Embase databases using the following search string on 31 January 2025: (child* OR paediatric* OR perinat* OR neonat* OR newborn* OR infan* OR baby OR babies OR toddler* OR juvenil* OR adolescen*) AND (discomfort* OR comfort* AND [fever OR pyrexia OR hyperthermia OR temperature OR febrile OR feverish OR body temperature]). The search was limited to articles published in English up to 31 January 2025.

### 2.2 Study selection

Studies were eligible for inclusion if they met the following criteria: (i) conducted on children aged 29 days to 18 years; (ii) assessed fever‐associated discomfort as a primary or secondary outcome; (iii) evaluated pharmacological or nonpharmacological interventions for discomfort relief; and (iv) were randomized controlled trials (RCTs) or observational studies. Exclusion criteria included: (i) studies focusing on fever management without assessing discomfort; (ii) studies conducted on adults or neonates; and (iii) studies with a sample size of fewer than 10 participants. Given their known high heterogeneity, definition of fever‐associated discomfort and methods employed for its evaluation were not considered for study inclusion or exclusion and were retained as provided by the original reports.^3^ Two independent reviewers initially screened the titles and abstracts of identified records. Full‐text articles of potentially relevant studies were then retrieved and evaluated for eligibility. Discrepancies were resolved through discussion with a third reviewer. Unlike the preregistered protocol, we decided to include not only studies with children older than 6 months but also those with children older than 28 days, to keep the research as comprehensive as possible.

### 2.3 Data extraction, quality assessment and endpoints

From each included study, the following data were extracted using a predefined data collection form: first author, year of publication, country, study design, sample size, setting, fever definition, enrolled population, details of the intervention (including type, dosage and administration method), methods used to assess discomfort and the main outcomes reported. The quality of RCTs was assessed using the Cochrane Risk of Bias tool, whereas the STROBE statement was applied to evaluate observational studies.^7^, ^8^

For each study, all outcomes related to discomfort relief and adverse effects were extracted, regardless of the measurement method or time point, as long as they were reported in relation to fever‐associated discomfort. When multiple measures or time points were available for the same outcome, we prioritized validated scales and outcomes reported at the final time point of follow‐up. If discomfort was reported both as a composite and as individual symptoms, both were considered. No outcomes were excluded based on the direction or significance of results. If relevant information (e.g., outcome definitions, time points or adverse events) was missing, we recorded it as not reported and made no assumptions about the missing data. No authors were contacted for clarification, and no imputation was performed.

The included studies were independently categorized based on the type of fever management strategy evaluated, including different antipyretic interventions and their comparative efficacy in paediatric populations. Specific data on discomfort assessment methods and definitions were also collected to evaluate consistency across studies. Both data extraction and quality assessment were performed independently by 2 reviewers and disagreement were addressed through discussion with a third reviewer.

The primary study endpoint was to compare different fever management strategies in terms of effectiveness to reduce discomfort. The secondary endpoint was to assess any potential adverse effects of the interventions. No statistical conversions or transformations of reported data (e.g., from median to mean) were performed due to variability in outcome formats.

Study characteristics and results were summarized in Table 1. Risk of bias assessments were visually displayed using traffic light plots (Figures S2 and S3), and the study selection process was illustrated using a PRISMA flow diagram (Figure S1).

**TABLE 1:** Included studies on fever‐associated discomfort management.

Author (year)^ref^	Country	Study design	Population	Intervention	Discomfort assessment	Key findings
Agbolosu *et al*. (1997)^9^	Malawi	RCT	80 children (6–54 months) with fever (38.5–40°C) due to URTI/malaria	Tepid sponging (repeated until temperature dropped to <38.5 °C) *vs*. paracetamol syrup by mouth at the beginning of the study (15 mg/kg)	Secondary outcome; evaluated by physicians via clinical parameters (convulsions, crying, irritability, vomiting, shivering)	Paracetamol significantly reduced fever; no significant difference in discomfort between groups.
Autret *et al*. (1997)^10^	France	Multicentre open RCT	351 children (6–24 months) with rectal temperature ≥39°C	Ibuprofen (7.5 mg/kg) *vs*. aspirin (10 mg/kg) *vs*. paracetamol (10 mg/kg). One dose was given, and no additional doses were administered in the next 6 h; further doses were allowed if necessary	Primary outcome; evaluated by physicians via pain/behaviour scales	Ibuprofen improved comfort more effectively than aspirin or paracetamol after 6 h.
Charde *et al*. (2023)^11^	India	Prospective observational	108 children (6 months–18 years) with fever/pain	Paracetamol (15 mg/kg/dose 4 times a day) *vs*. ibuprofen (10 mg/kg/dose 3 times a day) *vs*. combination of both	Primary outcome; evaluated by physicians using FLACC and visual analogue scales (pain as proxy for discomfort)	Combination therapy provided superior pain relief at 4 and 48 h compared to monotherapy.
Chetak *et al*. (2017)^12^	India	RCT	500 children (6 months–12 years) with axillary temperature >99°F (37.2°C)	Paracetamol (15 mg/kg) *vs*. paracetamol + tepid sponging	Secondary outcome; evaluated by physicians via clinical parameters (chills, goosebumps, irritability)	Tepid sponging added to paracetamol increased child discomfort.
Chiappini *et al*. (2023)^13^	Italy	Prospective observational	172 febrile children (29 days–18 years) in emergency department	Paracetamol (syrup or drops, children <3 months 10 mg/kg; children >3 months 15 mg/kg every 6 h)	Primary outcome; evaluated by nurses/parents via direct observation (sleep, appetite, motor activity, mood)	Paracetamol was associated with a reduction in discomfort (e.g., fatigue, restlessness) by 60 min post‐administration. Persistent fever correlated with higher initial discomfort.
Corrard *et al*. (2017)^14^	France	Prospective observational	200 febrile and 200 nonfebrile children (mean age ~19 months)	Paracetamol (mean dosage 14.8 mg/kg)	Primary outcome; discomfort assessed via caregiver‐reported sickness behaviour (irritability, crying, facial pain expressions)	Paracetamol was associated with an improvement in sickness behaviour (proxy for discomfort) in febrile children (*P* < .001), independent of fever severity.
Hay *et al*. (2009)^15^	UK	Blinded RCT	156 children (6 months–6 years) with axillary temperature ≥37.8°C	Paracetamol (15 mg/kg, every 4–6 h) *vs*. ibuprofen (10 mg/kg, every 6–8 h) *vs*. combination of both	Primary outcome; evaluated by nurses/parents via clinical parameters (pain/distress, crying)	No significant improvement in discomfort across treatment groups.
Polidori *et al*. (1993)^16^	Italy	Nonblinded RCT	110 children (3–6 years) with URTI‐related fever	Nimesulide (1.5 mg/kg, 3 times a day) *vs*. paracetamol syrup (10 mg/kg 4 times a day)	Secondary outcome; evaluated by physicians via a 4‐point scale	Nimesulide and paracetamol showed equivalent efficacy in reducing fever‐associated discomfort.

Abbreviations: FLACC, Face, Legs, Activity, Cry, and Consolability; RCT, randomized controlled trial; URTI, upper respiratory tract infection.

Due to the limited number of studies and methodological heterogeneity, no subgroup analyses or meta‐regressions were performed. Potential sources of heterogeneity were examined descriptively.

No sensitivity analyses were conducted.

## 3 RESULTS

Using the search strings, 3300 papers were retrieved in total. Of these, the full text of 113 articles was assessed. After excluding 97 papers that did not include original cases, or were not paediatric or not related to fever, 16 papers remained. An additional 8 papers were excluded because, although potentially relevant, they did not consider the outcomes of interest for this analysis (details and references are provided in Table S1). Finally, 8 studies evaluating the management of fever‐related discomfort in paediatric populations were included in this systematic review (Table 1).

### 3.1 Study characteristics

The systematic review included 8 studies published between 1993 and 2023, encompassing 1877 paediatric participants from diverse geographic regions (Malawi, France, India, Italy, UK).^9^, ^10^, ^11^, ^12^, ^13^, ^14^, ^15^, ^16^ Study designs comprised 5 RCTs and 3 observational studies (2 prospective and ‐one multicentre), with sample sizes ranging from 80 to 500 participants. Paediatric populations varied widely. Age ranges spanned infancy (6 months) to adolescence (18 years), with fever thresholds defined as axillary temperatures ≥37.8°C,^15^ or rectal temperatures ≥39°C.^10^

### 3.2 Evaluation of discomfort

Discomfort was explicitly defined in 5 studies, primarily through behavioural and clinical indicators. Agbolosu *et al*. and Chetak *et al*. operationalized discomfort as “convulsions, crying, irritability, vomiting, shivering” and “chills/goosebumps”,^9^, ^12^ while Chiappini *et al*. adopted a multidimensional framework encompassing “sleep‐wake cycle disruptions, reduced appetite, altered motor activity, mood changes, and facial expressions”.^13^ Corrard *et al*. focused on caregiver‐reported “sickness behaviour”, including irritability, crying and pain‐related facial expressions.^14^ In contrast, 4 studies conflated discomfort with pain,^11^, ^16^ or provided no explicit definition.^10^

Discomfort assessment methods were heterogeneous: 5 studies relied on clinician‐reported clinical parameters (e.g., crying, vomiting), 3 utilized validated tools such as the Face, Legs, Activity, Cry, and Consolability (FLACC) scale, visual analogue scale or a 4‐point behavioural scale,^11^, ^15^, ^16^ and 2 incorporated caregiver or parental observations.^13^, ^14^ Only ‐one study employed direct observational methods to track symptom changes pre‐ and postintervention.^13^ No study incorporated objective or possible physiological markers for discomfort (e.g., heart rate, cortisol).

### 3.3 Interventions and management

Paracetamol (10 mg/kg/dose) showed efficacy in reducing behavioural markers of discomfort, such as restlessness and irritability, within 60 min of administration,^13^ and improved caregiver‐reported sickness behaviour independent of fever severity.^14^ However, Hay *et al*. found no significant discomfort reduction with paracetamol monotherapy.^15^ In 2 studies, oral ibuprofen (7.5–10 mg/kg/dose) outperformed both oral paracetamol (10 mg/kg/dose) and aspirin (10 mg/kg/dose) in improving comfort: Autret *et al*. concluded that ibuprofen is more effective than paracetamol and aspirin in relieving discomfort 6 h after administration, but it presents more side effects.^10^

Charde *et al*., in a population study conducted between 6 months and 18 years, concluded that ibuprofen should be initially used for the treatment of fever. However, if the fever persists or is associated with pain, the combination with paracetamol would be the optimal choice, since they reported significant superior pain/discomfort relief with combined paracetamol and ibuprofen in faster pain relief at 4 h and at 48 h.^11^ Hay *et al*. found no additive benefits for combination therapy.^15^ Nimesulide, less commonly studied and approved in paediatric age, exhibited equivalent efficacy to paracetamol in reducing fever‐associated discomfort.^16^

Tepid sponging, assessed in 3 RCTs, effectively reduced fever but failed to alleviate discomfort. Agbolosu *et al*. compared the effectiveness of sponging with paracetamol. It was concluded that paracetamol is more effective in reducing body temperature, while sponging is most effective within the first 30 min of application, without maintaining a low temperature in the long term. Agbolosu *et al*. reported no difference in discomfort between sponging and paracetamol,^9^ while Chetak *et al*. observed “increased discomfort” (e.g., chills, irritability) when antipyretics were combined with sponging, rather than using paracetamol alone, and for this reason discourages the use of tepid sponging while treating fever.^12^

### 3.4 Safety

Studies reporting on paracetamol mainly reported no or mild adverse events. Chiappini *et al*. observed no adverse events related to paracetamol during the study period,^13^ while Polidori *et al*. reported adverse events, specifically vomiting, diarrhoea, or urticaria, in 9 patients receiving paracetamol compared to 5 patients treated with nimesulide.^16^ Charde *et al*. found that combination therapy with paracetamol and ibuprofen was associated with minimal adverse effects and no significant disturbances in biochemical parameters.^11^

Autret *et al*. noted that adverse events, especially gastrointestinal side effects, were significantly more frequent in the ibuprofen group (with 9 patients reporting 13 events) compared to the paracetamol group, and children in the ibuprofen group also exhibited a higher incidence of disgust or refusal of the drug than those receiving paracetamol.^10^ Hay *et al*. similarly reported that, although diarrhoea and vomiting were evenly distributed among treatment groups, a small subset of children, specifically 3 patients in the ibuprofen group, required hospital admission for serious adverse events.^15^ However, independent review determined that none of these events were directly related to the study interventions.

Chetak *et al*., even not including safety outcomes in their study, observed that adding tepid sponging to antipyretic treatment increased discomfort in children, manifested by signs such as chills and irritability, thus representing a possible adverse effect inherent to the procedure.^12^ Agbolosu *et al*. and Corrard *et al*. did not evaluate safety outcomes related to nonpharmacological methods. None of the studies reported life‐threatening or severe adverse events.^9^, ^14^

### 3.5 Quality of the studies

Among the 5 randomized controlled trials assessing interventions for fever‐related discomfort, 60% (*n* = 3) were identified as having some concerns regarding overall risk of bias (Figure S2). One trial was assessed as having a low risk of bias, while another was found to have a high risk of bias. Regarding the randomization process, ‐one study exhibited a low risk of bias, another showed a high risk of bias and the remaining 3 had some concerns. The risk of bias was low in the sections on deviations from intended interventions, missing outcome data and measurement of the outcome. Concerning the selection of reported results, 3 studies raised some concerns. All RCTs followed an intention‐to‐treat approach.

Regarding the 3 observational studies evaluating interventions for fever‐related discomfort, all showed a low risk of bias based on title and abstract (Figure S3). In the introduction section, a low risk of bias was identified across all studies, except for ‐one study that raised some concerns related to objectives. The evaluation of study methods, divided into study design, setting, participants, variables, data sources/measurement, bias, study size, quantitative variables and statistical methods, revealed that all 3 studies presented some concerns. In particular, ‐one study demonstrated a high risk of bias related to study size. The assessment of the results section, categorized into participants, descriptive data, outcome data, main results and additional analyses, indicated that only ‐one study had a low risk of bias across all these domains, while the other 2 studies exhibited some concerns. Specifically, some concerns were identified in participants and additional analyses. However, outcome data and main results were consistently judged to have a low risk of bias in all 3 studies. The 3 studies were assessed as having a low risk of bias in the reporting of key results and interpretation. Nevertheless, one study showed some concerns in limitations while 2 studies presented some concerns regarding generalizability. Finally, the evaluation of funding sources found a low risk of bias across all 3 observational studies.

## 4 DISCUSSION

Fever remains one of the most common clinical conditions in children, often leading to significant parental concern and healthcare utilization. This systematic review is the first, to our knowledge, to summarize evidence on effectiveness and safety of methods to manage discomfort in children with fever. The three main findings of this study are the following: (i) overall, a very limited number of studies with relevant heterogeneity exists on this topic; (ii) pharmacological approaches are likely to be effective and safe to reduce discomfort, but few studies have compared different drugs; and (iii) physical methods are likely to be ineffective.

It is widely recognized that fever is a physiological response to infection rather than a disease itself.^17^ However, management strategies by caregivers and sometimes, also by healthcare providers, often prioritize temperature reduction rather than symptom relief, despite international guidelines advocating for a shift in treatment focus.^14^, ^18^, ^19^ A key finding of this review is the paucity of studies evaluating fever‐associated discomfort as a primary outcome. While multiple RCTs have assessed the efficacy of pharmacological and nonpharmacological interventions in lowering body temperature, as summarized in a recently published meta‐analysis that included 31 RCTs addressing the effects of strategies to reduce body temperature in children with fever,^20^ we identified only 5 RCT, which considered as outcome the reduction of discomfort. This gap in research further supports a misalignment between clinical practice guidelines and the available scientific evidence.^21^, ^22^ Furthermore, available studies differed not only for the tested interventions, but also in the tools employed to measure discomfort. While 5 studies explicitly defined discomfort through behavioural and clinical parameters, such as crying, irritability, shivering, altered sleep–wake cycles and caregiver‐reported sickness behaviour,^9^, ^12^, ^15^ the remaining articles conflated discomfort with pain or provided no clear‐cut definition.^10^, ^11^, ^16^ For instance, Charde *et al*. equated pain with discomfort, using the FLACC and visual analogue scales,^11^ while Polidori *et al*. relied on a nonvalidated 4‐point clinician‐rated scale.^16^ This variability complicates cross‐study comparisons and reflects broader inconsistencies in clinical practice, where subjective interpretations might drive treatment decisions.

Regarding pharmacological interventions, paracetamol and nonsteroidal anti‐inflammatory drugs, such as ibuprofen, were the most commonly studied. While one study found ibuprofen to be more effective in relieving discomfort,^10^ 2 other studies reported no significant difference between ibuprofen or nimesulide and paracetamol.^15^, ^16^ The quality of the studies varied, with one study having a high risk of bias,^16^ another having a low risk of bias^15^ and the third raising some concerns.^10^ Furthermore, the dosages of both paracetamol and ibuprofen varied across studies. For instance, Autret *et al*. employed a dose of 10 mg/kg, which is lower than the routinely recommended dose of 15 mg/kg.^10^, ^23^ Finally, an observational study suggested that combination therapy with paracetamol and ibuprofen may provide superior relief compared to monotherapy.^11^ Given these mixed findings, further high‐quality trials are needed to determine the most effective pharmacological approach for managing discomfort. The use of paracetamol and ibuprofen was associated in all trials with no or mild adverse events, confirming their well‐known safety profile. By contrast, their use to manage fever associated discomfort should be cautiously considered in patients at higher risk of adverse events (e.g., kidney damage associated to the use of nonsteroidal anti‐inflammatory drugs in children with relevant dehydration). Of note, nimesulide is nowadays not recommended for children due to the potential side effects.^24^, ^25^

The findings of this review have potential implications for clinical practice and research. Given the emphasis on temperature reduction in current studies, which might partially depend on the widespread beliefs on its negative role,^21^, ^26^, ^27^ there is a critical need for future research to prioritize discomfort relief as an outcome of interest. The development of standardized, validated discomfort assessment tools would facilitate more robust comparisons across studies and help align research with guideline recommendations.^3^ Additionally, further trials assessing the efficacy and safety of pharmacological and nonpharmacological interventions specifically for discomfort relief are warranted.

This review also has limitations. The heterogeneity in study designs, outcome definitions, discomfort assessment methods and low number of high‐quality studies limits the comparability of results across studies and the strength of the available evidence. Many included studies focused on fever reduction rather than discomfort relief as a primary endpoint, leading to indirect inferences. Furthermore, the lack of standardized tools for measuring discomfort in febrile children remains a significant gap in the literature. This study also has strengths. First, it provides a comprehensive synthesis of available evidence on interventions for fever‐associated discomfort, an area that remains underexplored despite its clinical importance. The inclusion of both pharmacological and nonpharmacological interventions allows for a balanced perspective on their relative efficacy and safety. Additionally, adherence to PRISMA guidelines and the use of validated tools for quality assessment enhance the reliability of our findings.

In conclusion, it is well recognized that fever is usually more considered than pain.^26^, ^27^ This analysis suggests that also discomfort might be under‐evaluated compared to body temperature in managing children with fever. This review underscores the disconnect between guideline recommendations and available evidence in the management of febrile children. While antipyretics remain widely used, their impact on discomfort remains insufficiently studied. Future research should prioritize discomfort relief as a primary outcome, develop standardized assessment tools and explore both pharmacological and nonpharmacological interventions to optimize the management of febrile children.

### 4.1 Nomenclature of targets and ligands

Key protein targets and ligands in this article are hyperlinked to corresponding entries in http://www.guidetopharmacology.org, and are permanently archived in the Concise Guide to PHARMACOLOGY 2021/22.

## AUTHOR CONTRIBUTIONS

Antonio Corsello performed the literature search, data extraction and quality evaluation and wrote the first draft of the manuscript. Ilaria Alberti performed the literature search, data extraction and quality evaluation and wrote the first draft of the manuscript. Sara Farhanghi contributed to the study design and data interpretation and wrote the first draft of the manuscript. Alessia Bonetti contributed to the study design and data interpretation and reviewed the first draft of the manuscript. Silvia Garattini contributed to the study design and data interpretation and reviewed the first draft of the manuscript. Anna Comotti contributed to data interpretation, prepared the tables, provided input in her field of expertise and reviewed the first draft of the manuscript. Paola Marchisio contributed to the study design and data interpretation, provided input in her field of expertise and reviewed the first draft of the manuscript. Elena Chiappini contributed to the study design and data interpretation, provided input in her field of expertise and reviewed the first draft of the manuscript. Gregorio Paolo Milani conceptualized the study, contributed to data interpretation, reviewed the first draft of the manuscript and provided input in his field of expertise. All authors approved the final version of the manuscript.

## CONFLICT OF INTEREST STATEMENT

G.P.M. received an unrestricted grant from Reckitt Benckiser Healthcare SPA and one from Angelini SPA. Furthermore, he participated in Advisory Boards supported by unrestricted grants from Angelini Pharma.

## Supporting information


**FIGURE S1.** PRISMA flow diagram for systematic reviews.
**FIGURE S2.** Quality assessment of randomized controlled trials (Cochrane Risk of Bias tool). Results are given collectively (upper panel) and per single study (lower panel).
**FIGURE S3.** Quality assessment of observational studies (Strobe).
**TABLE S1.** Additional excluded studies with reason for exclusion.

## Data Availability

The data collection form, extracted data and risk of bias assessments are available upon reasonable request to the corresponding author.
