# Supplementary material for: Effectiveness and safety of interventions for fever‐associated discomfort in children: A systematic review
Source: Br J Clin Pharmacol. 2025 Aug 22;91(12):3323–9. doi: 10.1002/bcp.70203 (PMC12648366; doi:10.1002/bcp.70203)
Supplement: Supplementary file 1 — FIGURE S1. PRISMA flow diagram for systematic reviews. FIGURE S2. Quality assessment of randomized controlled trials (Cochrane Risk of Bias tool). Results are given collectively (upper panel) and per single study (lower panel). FIGURE S3. Quality assessment of observational studies (Strobe). TABLE S1. Additional excluded studies with reason for exclusion. [file BCP-91-3323-s001.docx]

**Supplementary online material**

**Supplementary Figure 1.** PRISMA flow diagram for systematic reviews.


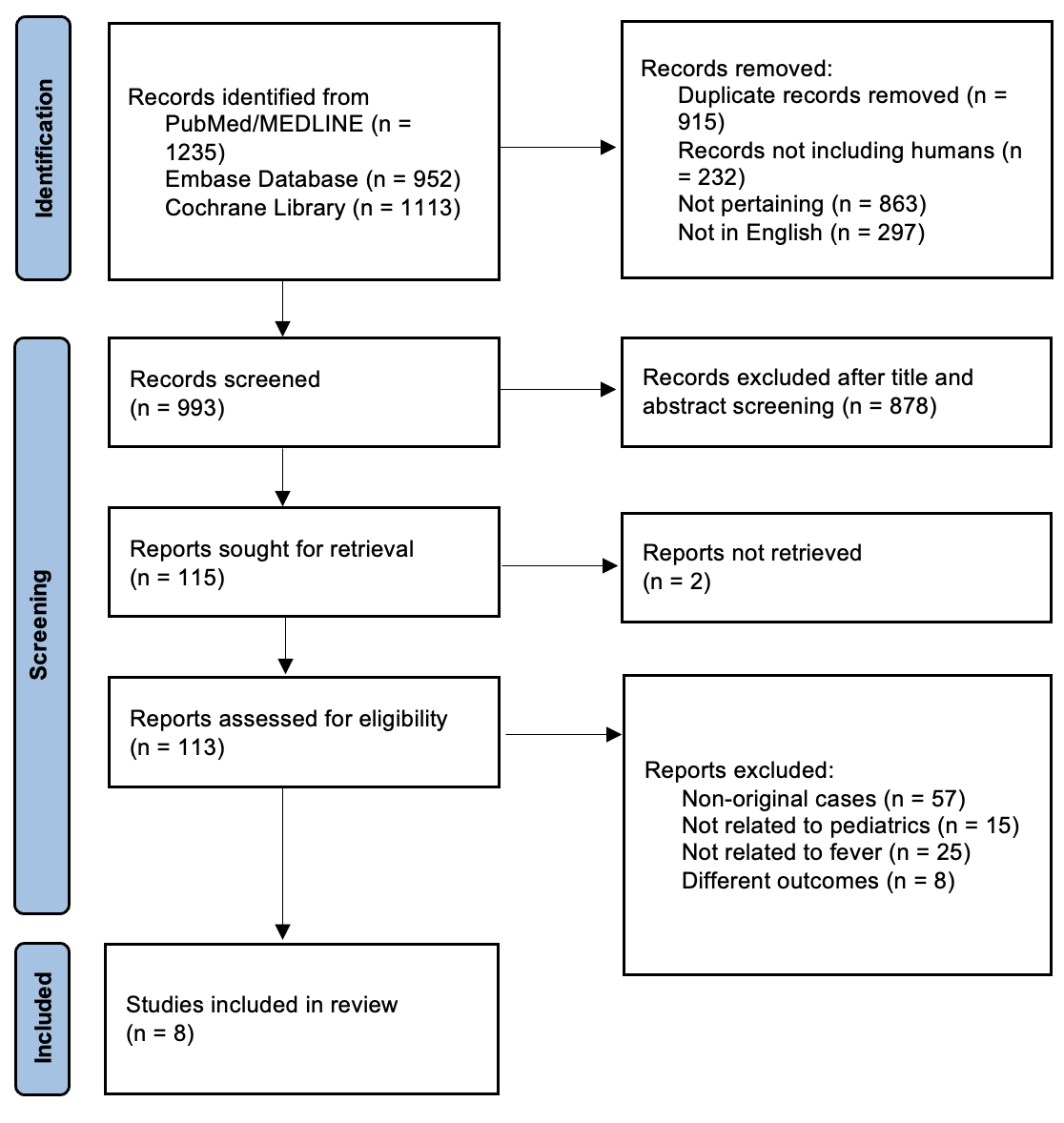


**Supplementary Figure 2.** Quality assessment of randomized controlled trials (Cochrane Risk of Bias tool). Results are given collectively (upper panel) and per single study (lower panel).

**
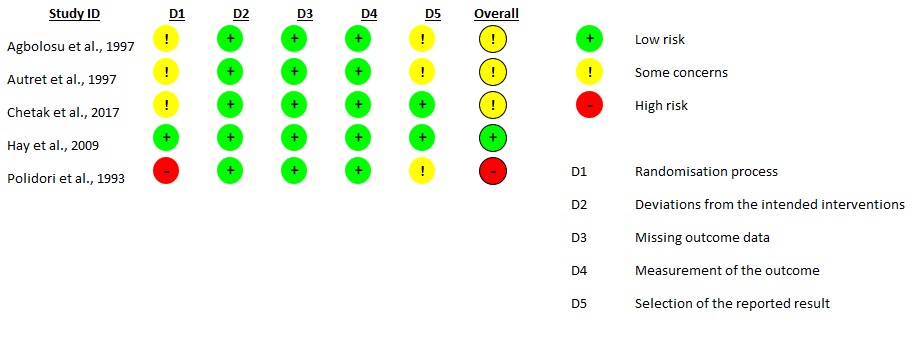
**

**Supplementary Figure 3.** Quality assessment of observational studies (Strobe).


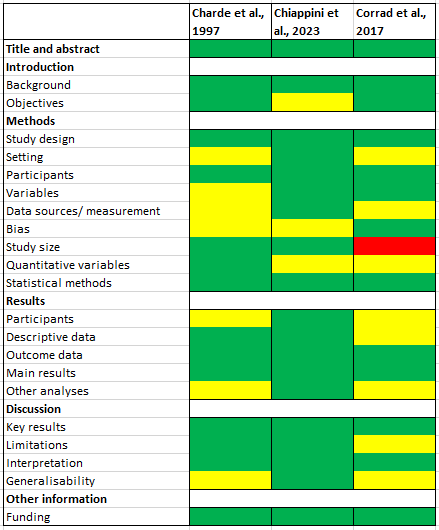


**Table S1. Additional excluded studies with reason for exclusion.**

| **Study** | **Year** | **Reason for exclusion** |
| --- | --- | --- |
| **Aluka T et al.^1^** | 2013 | Evaluated discomfort related to the sponge cooling procedure itself, not to fever. |
| **Alves JG et al.^2^** | 2008 | Measured discomfort associated with the procedure rather than with fever itself. |
| **Khaliq A et al.^3^** | 2019 | Reported discomfort pertaining to the intervention process, not to fever. |
| **Park Y et al.^4^** | 2021 | Focused on self-reported discomfort rather than clinically assessed fever-related symptoms. |
| **Peters MJ et al.^5^** | 2019 | Feasibility RCT: discussed discomfort qualitatively but did not measure it as a fever-related outcome. |
| **Sarrell EM et al.^6^** | 2006 | Assessed stress levels instead of discomfort. |
| **Sharber J.^7^** | 1997 | Investigated discomfort related to the intervention procedure rather than to fever. |
| **Thomas S et al.^8^** | 2009 | Investigated discomfort related to the intervention procedure rather than to fever. |

1. Aluka TM, Gyuse AN, Udonwa NE, Asibong UE, Meremikwu MM, Oyo-Ita A. Comparison of cold water sponging and acetaminophen in control of Fever among children attending a tertiary hospital in South Nigeria. J Family Med Prim Care. 2013;2(2):153-158. doi:10.4103/2249-4863.117409
2. Alves JG, Almeida ND, Almeida CD. Tepid sponging plus dipyrone versus dipyrone alone for reducing body temperature in febrile children. Sao Paulo Med J. 2008;126(2):107-111. doi:10.1590/s1516-31802008000200008
3. Khaliq A, Zeb R, Khan S, Ahmad I, Tahir M, Shah SIA. Comparison between antipyretic and cold sponging versus only antipyretic in treatment of fever in pediatrics age group. J Med Sci. 2019;27(1):3-6.
4. Park YR, Kim H, Park JA, et al. Comparative Analysis of Single and Combined Antipyretics Using Patient-Generated Health Data: Retrospective Observational Study. JMIR Mhealth Uhealth. 2021;9(5):e21668. Published 2021 May 26. doi:10.2196/21668
5. Peters MJ, Khan I, Woolfall K, et al. Different temperature thresholds for antipyretic intervention in critically ill children with fever due to infection: the FEVER feasibility RCT. Health Technol Assess. 2019;23(5):1-148. doi:10.3310/hta23050
6. Sarrell EM, Wielunsky E, Cohen HA. Antipyretic treatment in young children with fever: acetaminophen, ibuprofen, or both alternating in a randomized, double-blind study. Arch Pediatr Adolesc Med. 2006;160(2):197-202. doi:10.1001/archpedi.160.2.197
7. Sharber J. The efficacy of tepid sponge bathing to reduce fever in young children. Am J Emerg Med. 1997;15(2):188-192. doi:10.1016/s0735-6757(97)90099-1
8. Thomas S, Vijaykumar C, Naik R, Moses PD, Antonisamy B. Comparative effectiveness of tepid sponging and antipyretic drug versus only antipyretic drug in the management of fever among children: a randomized controlled trial. Indian Pediatr. 2009;46(2):133-136.
